# Integrating Molecular Network and Culture Media Variation to Explore the Production of Bioactive Metabolites by *Vibrio diabolicus* A1SM3

**DOI:** 10.3390/md17040196

**Published:** 2019-03-27

**Authors:** Natalia Conde-Martínez, Anelize Bauermeister, Alan Cesar Pilon, Norberto Peporine Lopes, Edisson Tello

**Affiliations:** 1 Bioscience Doctoral Program, Grupo de Investigación en Bioprospección, Faculty of Engineering, Universidad de La Sabana, Campus Puente del Común, Km 7, Autopista Norte de Bogotá, 250001 Chía, Colombia; nataliacoma@unisabana.edu.co; 2 Núcleo de Pesquisa em Produtos Naturais e Sintéticos (NPPNS), Faculdade de Ciências Farmacêuticas de Ribeirão Preto, Universidade de São Paulo, 14040-903 Ribeirão Preto/SP, Brazil; ane_qui@hotmail.com (A.B.); pilonac@gmail.com (A.C.P.); npelopes@fcfrp.usp.br (N.P.L.); 3 Departamento de Farmacologia, Instituto de Ciências Biomédicas, Universidade de São Paulo, 05508-900 São Paulo-SP, Brazil

**Keywords:** *Vibrio diabolicus*, molecular networking, 2,2-di(3-indolyl)-3-indolone, PHB, MS/MS, solar saltern

## Abstract

*Vibrio diabolicus* A1SM3 strain was isolated from a sediment sample from Manaure Solar Saltern in La Guajira and the produced crude extracts have shown antibacterial activity against methicillin-resistant *Staphylococcus aureus* and cytotoxic activity against human lung cell line. Thus, the aim of this research was to identify the main compound responsible for the biological activity observed and to systematically study how each carbon and nitrogen source in the growth media, and variation of the salinity, affect its production. For the characterization of the bioactive metabolites, 15 fractions obtained from *Vibrio diabolicus* A1SM3 crude extract were analyzed by HPLC-MS/MS and their activity was established. The bioactive fractions were dereplicated with Antibase and Marinlit databases, which combined with nuclear magnetic resonance (NMR) spectra and fragmentation by MS/MS, led to the identification of 2,2-di(3-indolyl)-3-indolone (isotrisindoline), an indole-derivative antibiotic, previously isolated from marine organisms. The influence of the variations of the culture media in isotrisindoline production was established by molecular network and MZmine showing that the media containing starch and peptone at 7% NaCl was the best culture media to produce it. Also, polyhydroxybutyrates (PHB) identification was established by MS/MS mainly in casamino acids media, contributing to the first report on PHB production by this strain.

## 1. Introduction

*Vibrionaceae* family (composed c.a. 128 species) is considered a Gram-negative and gamma-proteobacteria, ubiquitous in marine environments and has been spotted out for its genomic flexibility and mainly for their importance as pathogens [1]. Until 2011, only 93 bioactive compounds have been reported to be isolated from the *Vibrionaceae* family [2]. Recently, some studies revealed the potential of *Vibrionaceae* as a source of new and bioactive natural products [3]. Among them, *Vibrio spp*. are known to be the most representative species in the *Vibrionaceae* family (64 species) being characterized by the non-ribosomal peptides production and some hybrids synthesized by non-ribosomal peptide and polyketide synthases [2,4]. Another family of bioactive compounds that have been widely reported is the indole alkaloids which includes the turbomycin A and B [5], vibrindole A [6], trisindoline [7] and the bis-indolylmethane derivatives, presenting anticancer activities [2,8]. On the other hand, some marine species from *Vibrio* spp. have been studied for their potential to produce polyhydroxybutyrates (PHB), a biopolymer produced and accumulated by prokaryotes, widely studied for its biotechnological potential as an alternative solution to replace plastic due to their biodegradability properties [9].

Despite the biosynthetic potential of microorganisms to produce a variety of metabolites, several biological and analytical challenges still hamper their production, isolation, and identification [10]. The different forms of interaction between the strains and their surrounding environment (biotic and abiotic) determine the degree of metabolic diversification through a panoply of different biosynthetic pathways activation, including those related to the expression of cryptic genes—typically not expressed in normal laboratory conditions [11]. To address these limitations, the chemo-physiological conditions associated with different biosynthetic pathways expression have been studied by genomic strategies, metabolic engineering or system biology-based approaches such as OSMAC (one strain many compounds) and co-culture [12,13,14]. In OSMAC strategy, the different metabolites production is obtained by varying the growth parameters (biotic factors: temperature, pH, time, media composition, luminosity, etc.) using chemometric tools considering that within a sample space it is possible to correlate changes in the environment with different expressions levels of secondary metabolite biosynthesis [12].

Currently, microbial metabolomics and OSMAC studies are using advanced analytical techniques, especially LC-MS for the analysis of metabolic classes of interest [15]. Typically, large MS/MS fragmentation data sets generated by automated analysis are evaluated by chemometric tools or web services to reduce data complexity and promote clues about trends and similarities. Among prominent approaches, molecular networking (MN) seems to be a particularly effective tool for the processing of fragmentation data from tandem mass spectrometry (MS/MS), since it can visualize tendencies, group and identify compounds based on spectral similarity. As structurally related compounds share similar fragmentation spectra, the molecular network tends to form closely related agglomerates [10].

There are some emerging online platforms that enable analysis, interpretation, and dissemination of knowledge efficiently, reliably, and quickly based on the molecular network. The recent mass spectra consortium featured on the GNPS platform have played an important role on analysis of large datasets of tandem MS experiments from microbial extracts clustering of MS-related data visualization and consequently, leading to correlated biological information such as species grouping or information relative to bioactive compounds [16].

Considering that the antibacterial and cytotoxic activity of *Vibrio diabolicus* A1SM3 was established in our group [17], we proposed here a strategy combining different growth parameters with molecular network tool for rapid and reliable clustering and characterization of bioactive compounds. For this purpose, variation in carbon and nitrogen source and salinity of the initial culture medium were evaluated to establish how these changes enhance the production of 2,2-di(3-indolyl)-3-indolone named as isotrisindoline, an antibiotic indole alkaloid isolated from *Vibrio* species [18] and produced by the metagenomes genes of the sponge *Discodermia calyx* [19,20], and the PHB production, using a combined strategy of HPLC-MS/MS analysis and GNPS workflow.

## 2. Results

### 2.1. Growth of Vibrio diabolicus A1SM3 in Modified Culture Media

**Figure 1:**
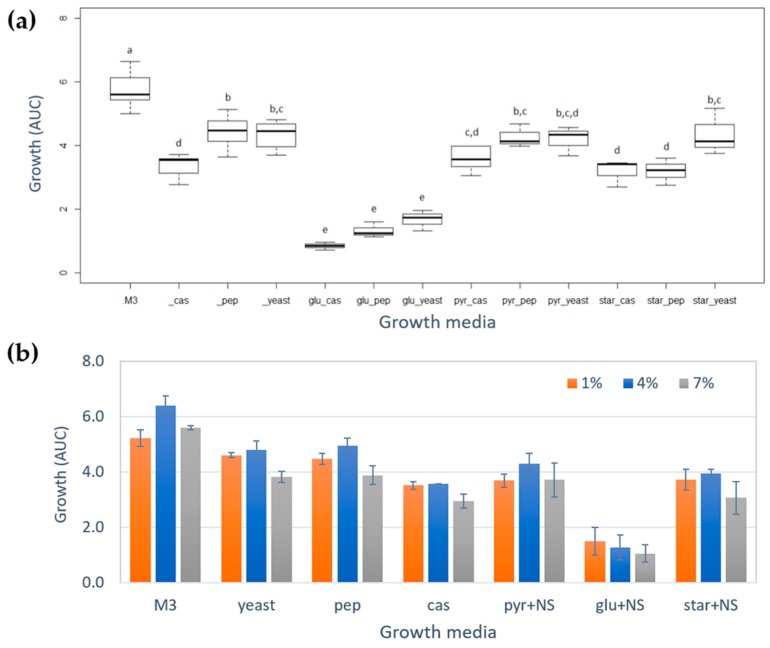
(**a**) Boxplot of growth of *Vibrio diabolicus* A1SM3 calculated as area under the curve (AUC) in each growth media. Glucose (glu), sodium pyruvate (pyr) and starch (star), casamino acids (cas), peptone (pep), and yeast extract. The results of AUC for each growth media are from the replicates and includes all the salinities tested. The boxes bearing different letters (a, b, c, d, and e) were significantly different according to the Tukey HSD test (*p* < 0.05). (**b**) Growth of *Vibrio diabolicus* A1SM3 calculated as AUC grouped by salinity and carbon source (as a binary mixture with the nitrogen source, NS), M3 medium and growth media with only nitrogen source (without carbon source), casamino acids (cas), peptone (pep), and yeast extract.

The initial culture medium (M3 medium) where the bioactive compounds were detected contain glucose (glu), starch (star), and sodium pyruvate (pyr) as carbon sources, and yeast extract, peptone (pep) and casamino acids (cas) as nitrogen sources [17]. From this initial composition, 12 modified culture media were proposed (Table 1) to establish how each of these nutrient sources affect the growth and the metabolic profile of *Vibrio diabolicus* A1SM3. The differences in the bacterial growth of *Vibrio diabolicus* A1SM3 in the different modified media (Supplementary Figure S1), were compared by the area under the curve (AUC) calculated for each culture at each salinity. From two-way ANOVA analysis was established a significant difference in the AUC between cultures (level: cultures; F = 70.89, *p* = 2 × 10^−16^) and the results from Tukey HSD post-hoc test showed that the growth in the media with glucose as the sole carbon source was significantly lower compared to the growth in the media with sodium pyruvate and starch, and also compared with the cultures that only have a nitrogen source (Figure 1a).

This behavior revealed that the presence of glucose had some negative effect on *Vibrio diabolicus* A1SM3 growth under these conditions. In addition, the best medium to grow this strain was the initial M3 medium (Figure 1a).

Additionally, from two-way ANOVA considering the media and the salinity, was established that the growth of *Vibrio diabolicus* A1SM3 was significantly affected (level: salinity; F = 19.42, *p* = 1.4 × 10^−6^) by the salinities tested (Figure 1b). In Figure 1b was evidenced the differences in the growth in the modified media by salinity. For M3 media, the higher growth was observed at 4% of NaCl.

### 2.2. Study of the Metabolic Profile of Vibrio diabolicus A1SM3 in the Modified Culture Media

**Figure 2:**
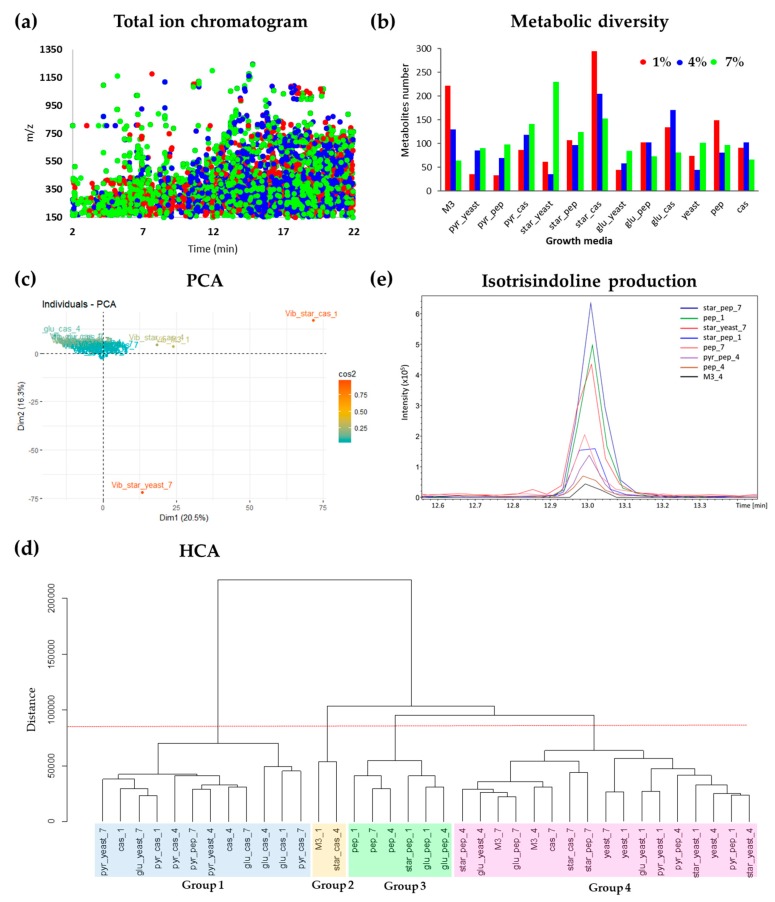
Metabolic profile description of the crude extracts produced by *Vibrio diabolicus* A1SM3 in modified culture media considering the HPLC-MS analyses. (**a**) Total ion chromatogram. (**b**) Metabolic diversity showing the number of metabolites produced in the mixture design. (**c**) Principal component analysis (PCA) showing the outlier samples star_yeast_7 and star_cas_1 (Supplementary Table S1). (**d**) Hierarchical clustering analysis (HCA) without the outliers indicated in PCA, showing a clustering mainly by nitrogen sources. (**e**) Isotrisindoline peak (*m*/*z* 364) in the crude extracts of different modified culture media compared with M3 medium at 4%.

The metabolic profile produced by *Vibrio diabolicus* A1SM3 in the modified culture media was investigated here by analyzing the crude extracts by HPLC-MS/MS. The data were treated in MZmine software and the peak area was used for this investigation. Figure 2a shows the ion (*m*/*z*) dispersion by the retention time and it is possible to observe clearly that most metabolites have less polarity characteristic. Moreover, most of the ions were up to about 800 (*m*/*z*), and only a few ions were observed above 1000 (*m*/*z*). Figure 2b shows that the lower metabolic diversity production was observed in media with yeast extract: pyr_yeast_1 (35 metabolites) and star_yeast_4 (36 metabolites), except for pyr_pep_1 (33 metabolites) which could be justified by the presence of the carbon source sodium pyruvate, which apparently is also related with few metabolites production. When considering the mixture of all the components in M3 medium, it was observed that the salinity showed an inversely proportional influence on metabolites production, the increase of salinity lead to a decrease of metabolic diversity.

On the other hand, greater metabolic diversity production was observed in the growth media containing starch plus casamino acids at 1% NaCl (295 metabolites), followed by starch plus yeast extract at 7% NaCl (230 metabolites) and M3 at 1% NaCl (222 metabolites). Therefore, comparing the same carbon source, the casamino acids was the most important parameter to induce metabolic diversity production. Interestingly, starch plus yeast extract combination at 7% NaCl, *Vibrio diabolicus* A1SM3 also produced a great number of metabolites (230), however, it was not possible to correlate this result with any variable evaluated in the culture media. In general, it seems that the nitrogen source was the most influential component in metabolites production. In principal component analysis (PCA) (Figure 2c) the crude extracts obtained from star_yeast_7 and star_cas_1 were the samples with less correlation with the others (Supplementary Figure S2). Therefore, these samples were considered as outliers and were removed from the table to build a hierarchical clustering analysis (HCA) (Figure 2d). In Figure 2b it is possible to evidence the clustering of the cultures in four main groups. Group 1 are formed mainly by cultures containing as nitrogen source casamino acids, as well as, group 3 formed only by growth media containing peptone and group 4 formed mainly by cultures containing yeast extract, which statistically support the hypothesis that the nitrogen sources are the most important nutrient evaluated in the culture media metabolic diversity by *Vibrio diabolicus* A1SM3. Additionally, no grouping by salinity was evidenced, meaning that, even though the salinity influences the growth of the strain, no relationship was observed between the variations in the metabolic profile and the salinities tested (Figure 2d).

### 2.3. Cytotoxic Activity and Dereplication of the Fractions from Vibrio diabolicus A1SM3 Grown in M3 Medium

Considering that M3 medium at 4% NaCl was the initial medium to growth *Vibrio diabolicus* A1SM3 and its crude extract showed the bioactivities, 4L of culture was extracted with ethyl acetate and the crude extract was fractionated by column chromatography using as eluent solvent a gradient from n-hexana:ethyl acetate (7:3) to ethyl acetate:methanol (9:1), obtaining 15 grouped subfractions according to the TLC profile (Supplementary Table S2). The cytotoxic activity of 10 of these fractions was evaluated against two human cancer cell lines and one non-tumor cell line (control toxicity) were used. The results showed that fractions F4 and F6 were slightly cytotoxic against the human cervix epithelial cancer cell line, SiHa (ATCC^®^ HTB – 35 ^TM^) with IC_50_ of >100 µg/mL and 80 µg/mL, respectively. On the other hand, fraction F5 displayed a stronger activity with an IC_50_ of 28 µg/mL against the same cell line. In addition, none of these fractions showed inhibition of the non-tumor cell line (IC_50_ >100 µg/mL).

**Figure 3:**
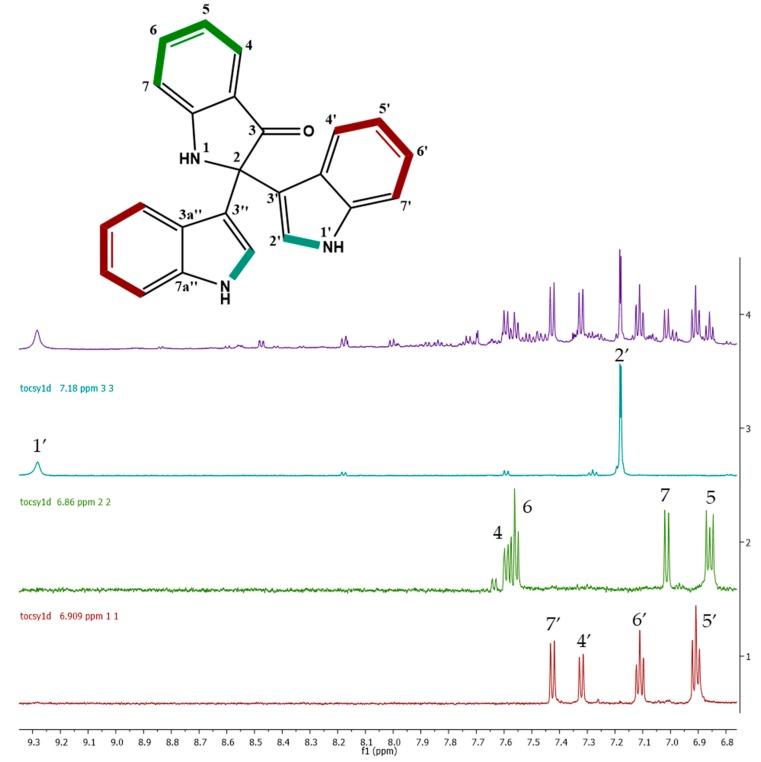
Mono dimensional ^1^H-TOCSY experiment (600 MHz, CD_3_CN) at 7.18, 6.909, and 6.86 ppm signals for F5 fraction. Bold bonds correspond to the spin systems established in isotrisindoline.

According to the analysis of the chromatographic profiles of these three cytotoxic fractions, F5 showed the most intense peak at 12.9 min (Supplementary Figure S3), which were also found in F6 with less intensity, and in F4 as a small peak. These results could be correlated with the cytotoxic assay, due to the stronger activity showed by F5 compared to F6 and F4. This peak showed *m*/*z* 364.1461 [M + H]^+^ and the molecular formula was determined as C_24_H_18_N_3_O^+^ (calculated mass of 364.1450 Da, 3 ppm). The dereplication analysis for ion *m*/*z* 364.1461 using Antibase and MarinLit databases showed a match for trisindoline and isotrisindoline (2,2-di(3-indolyl)-3-indolone), an antibiotic indole-trimer alkaloids both previously isolated from associated-bacterium *Vibrio* sp. from marine sponge *Hyrtios altum* [7] and from *Vibrio parahaemolyticus* [5,6]. The MS/MS spectrum (Supplementary Figure S3) showed that the fragmentation of *m*/*z* 364.1458 resulted in the fragment *m*/*z* 336 as the most intense fragment ion, corresponding to a neutral carbon monoxide loss (C=O). The ion at *m*/*z* 247 corresponds to a neutral loss of 117 Da, representing the cleave of an open indole moiety (C_8_H_7_N) which was corroborated by the ion *m*/*z* 247.0844 (calculated for C_16_H_11_N_2_O^+^; 247.0866 Da, 8.7ppm) in HR-ESI-MS spectra. Finally, the ion at *m*/*z* 219 corresponds to the loss of the indole moiety from fragment *m*/*z* 336. Considering that F5 was an impure fraction, a mono-dimensional ^1^H TOCSY (600 MHz, CD_3_CN) experiment at 7.18, 6.909, and 6.86 ppm signals were performed, and three spin systems were established (Figure 3, bold bond). The first one corresponding to the aromatic ring of the indole moieties from H-4′ to H-7′. The second one corresponds to the aromatic ring of an oxy-indole moiety from H-4 to H-7 and, finally the coupling between the NH and H-2′. This information, combined with HSQC, HSQC-TOCSY, and HMBC experiment (600 MHz, CD_3_CN), and the reported in the literature [6,20] led to confirm the identification of this compound as isotrisindoline, the major component in fraction F5 and the responsible for their cytotoxic activity (Supplementary Table S3 and Figure S4). The signals on the NMR data that led to establish the difference between the isotrisindoline and the trisindoline were the corresponding to the carbons in the oxy-indole ring, mainly the signals for the quaternary carbons 3a and 7a, followed by the signals of carbons 5 and 6 (Supplementary Table S3).

### 2.4. Molecular Networking and Isotrisindoline Production

HPLC-MS/MS data obtained from the 54 extracts from the modified culture media, 15 fractions, and 13 non-inoculated media controls, were converted to mzXML format, uploaded to GNPS web-platform and used to build a molecular network considering the online workflow. This tool clustered MS/MS spectra based on the similarity between them. After that, the data was exported and visualized in Cytoscape [21].

**Figure 4:**
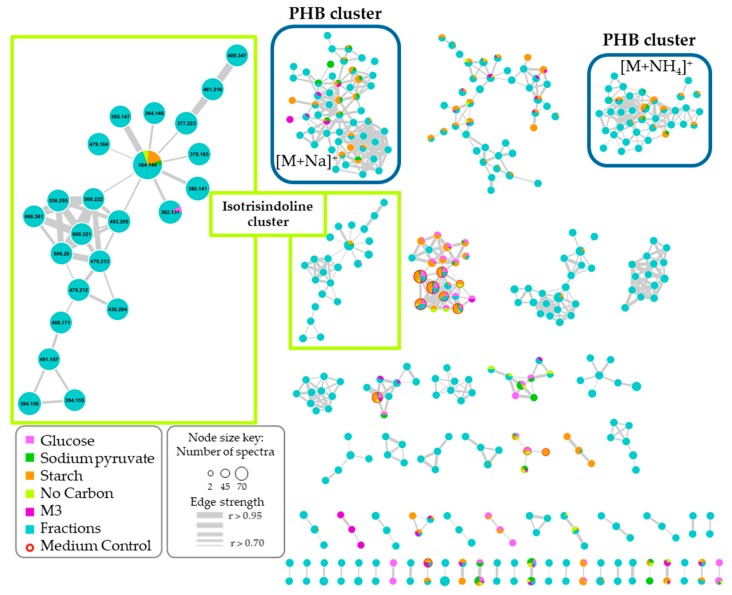
Molecular network for *Vibrio diabolicus* A1SM3 culture extracts in different growth media and including the fractions from the crude extract in the M3 growth medium. Each node is associated with an ion found in at least one of the growth cultures. The modified culture media were colored by carbon source as follows; glucose (pink), sodium pyruvate (dark green), and starch (orange). Nodes found in the M3 medium are colored as purple and modified culture media with only nitrogen source (no carbon) are light green. The nodes found in fractions are in blue and the nodes also found in the extract of the non-inoculated medium control are circled in red. The cluster associated with isotrisindoline is marked in a light green square and PHB cluster in a blue square.

A total of 705 nodes representing unique spectra (removing solvent blank nodes) were grouped in 57 clusters conformed with at least 2 nodes (Figure 4). No matches were found by GNPS library (reference compounds present in the platform), which may suggest new metabolites presence, or at least, similar compounds lacking in the GNPS library. This network was colored according to the carbon source used in the growth media and differencing the fractions (light blue) from them. This color-coding was useful to visualize the general production of metabolites under the specific conditions and which of them were also found in the fractions. In general, it was observed a correlation between the metabolites production (as nodes in the network) with the bacterial growth behavior aforementioned.

Once the ion associated with the isotrisindoline molecule (*m*/*z* 364.1450) was located in the molecular network, its presence in the cultures containing as sole carbon source, starch, and sodium pyruvate was established. Additionally, it was also detected in the growth media with only a nitrogen source as a nutrient source, peptone, and yeast extract. However, it was evidenced the lack of the M3 medium within this node. This is explained due to the low intensity and poor-quality of the MS/MS spectrum that correspond to the ion *m*/*z* 364.1450 in the raw data of M3 culture extracts which did not allow that GNPS algorithm to correctly grouped the MS/MS spectra from isotrisindoline in the node for this ion. Putting together this information with the peak area obtained in the aligned peak list from MZmine processing, it was possible to establish that the major peak area for isotrisindoline was in the cultures with starch plus peptone at 7% NaCl, followed by peptone at 1% and starch combined with yeast at 7% NaCl (Figure 2e). These results suggest that isotrisindoline production was higher in the cultures with starch and peptone at the three salinities tested.

It is important to mention that the non-inoculated media were subjected to the same incubation period and extraction procedures. The search of the ion corresponding to isotrisindoline in the HPLC-MS/MS data of non-inoculated media extracts showed that this ion was not present, confirming that it is not an artifact from the medium. This proves that this compound was produced by *Vibrio diabolicus* A1SM3 and that is not generated only from media components during work-up procedures, as some authors have been described in the literature [2,5].

### 2.5. Polyhydroxybutyrates (PHB) Molecular Family

On the other hand, exploration of the vast information merged in the network and compared with literature and the Antibase database, it was possible to identify two clusters associated with the polyhydroxybutyrates (PHB) chemical family (Figure 4). These compounds are known to be the most common polyhydroxyalkanoates (PHA) produced by microorganisms under stress conditions, combined with an excess of carbon source, and accumulated as reserves of carbon and energy [22,23]. Additionally, some species from *Vibrio* spp. have been described as PHB producer strains [22,24,25,26]. In the network, despite to belong to the same chemical family, two clusters of nodes were associated with PHB, one cluster grouped the adducts with sodium [M + Na]^+^ and the other with ammonium [M + NH_4_]^+^.

**Figure 5:**
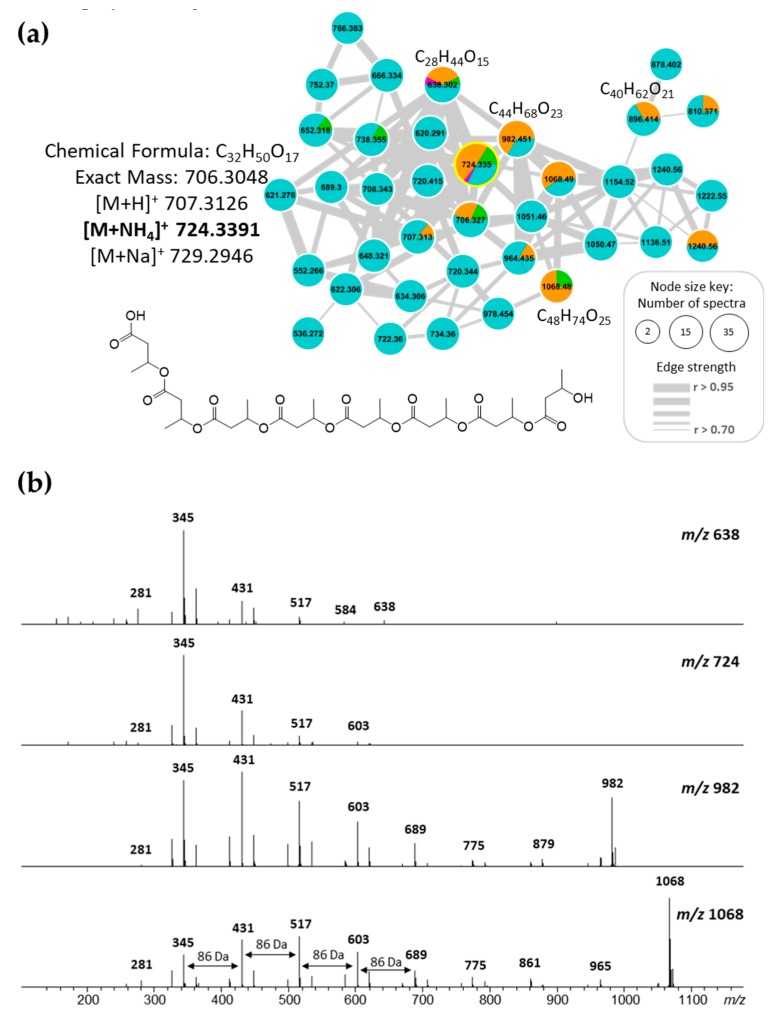
(**a**) PHB cluster of [M + NH_4_]^+^ adducts and molecular structure of PBH analog of the most representative node in the cluster. (**b**) MS/MS fragmentation pattern for most representative nodes in [M + NH_4_]^+^ adduct of PHB cluster.

The identification of PHB was established by the fragmentation pattern observed in MS/MS spectra of the nodes grouped in both clusters. As an example, the ion peak at *m*/*z* 724.3391 corresponding to [M + NH_4_]^+^ adduct, according to peaks ions *m*/*z* 707.3126 as [M+H]^+^ and *m*/*z* 729.2946 as [M + Na]^+^ presence, which matches with the molecular formula of C_32_H_50_O_17_ (calculated mass of 706.3048 Da) (Figure 5a). This pattern of adducts was observed in all nodes for both PHB clusters. The MS/MS spectra for both [M + Na]^+^ and [M+NH_4_]^+^ adducts revealed the successive loss of 86 Da corresponding to a fragment of molecular formula C_4_H_6_O_2_ which corresponds to the butyrate fragment in PHB polymer (Figure 5b).

**Figure 6:**
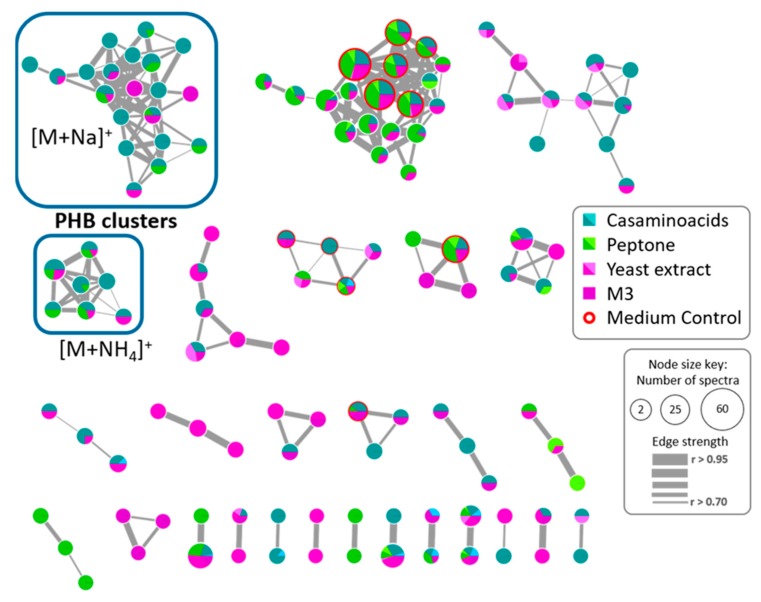
Molecular network for *Vibrio diabolicus* A1SM3 culture extracts in the modified culture media. The mixture design cultures by nitrogen source were colored as follows; casamino acids (blue), peptone (green), and yeast extract (pink). Nodes found in the M3 medium are colored as purple and the nodes found also in the extract of the non-inoculated medium control are circled in red. The lighter tones are associated with the growth media without any of the carbon sources. The PHB related clusters are in a blue square.

Considering that most of the nodes observed in the first network belong to the fractions, a new network was built considering only the MS/MS data of the modified culture media to explore the variation in PHB production by *Vibrio diabolicus* A1SM3 in the different growth media (Figure 6). This network allowed to establish that the PHB production was independent of the nitrogen sources (Figure 6). However, the presence of casamino acids stimulates the production of more PHB analogs. Considering the cluster for PHB with sodium [M + Na]^+^ (Figure 6), 90% of the nodes were present in cultures with casamino acids, furthermore, 45% were exclusively produced in the culture media containing casamino acids. In addition, the same proportion was maintained in the cluster for [M + NH_4_]^+^ adducts.

Additionally, it was observed that PHB was exclusively produced in the media with sodium pyruvate and starch as the sole carbon source, this could be correlated with the negative effect in the growth of the microorganism in the culture media containing glucose as the sole carbon source (Figure 1a). In addition, it was evidenced that the carbon source presence in the growth media was necessary to PHB production because no PHB nodes were observed in the cultures with only nitrogen as a nutrient source even though the microorganism grew comparable with the other growth media. This was correlated with the biological function of the PHB family in microorganisms as a reserve of carbon and energy source.

## 3. Discussion

Among the growth media tested at different salinities, it was possible to establish that the best growth of *Vibrio diabolicus* A1SM3 was at 4% NaCl, the same salinity of the pond from it was isolated in Manaure Solar Saltern. Additionally, it was found that the nitrogen source was the most relevant parameter affecting the metabolic profile, being the casamino acids the source that enhance the number of metabolites in the culture extracts. Among the carbon sources, the starch showed to be the only that affects positively the metabolic production. Although the salinity significantly affects the growth of the strain, no relationship between the salinity and the number of metabolites produced was evidenced.

The MS/MS analysis with GNPS allowed to visualize that there are many clusters from ions present only in the fractions, which means the analysis considering crude extracts could be leaving out many important ions that are in minor concentration or that does not have intense ionization. Therefore, we strongly suggest crude extracts fractionation before using in a molecular network or other metabolomic tools. Additionally, the development of MS/MS techniques and the evolution of the algorithms associated to this data are in constant improvement to enhance reproducibility and reliability under the same analysis parameters. This leads to consistent results when it comes to dealing with a biological process involving living organisms and their inherent variations related to their sensibility to environmental changes.

The strategy followed in this research led to isotrisindoline identification, a bioactive compound present in fraction F5 responsible for the cytotoxic activity against human cervix epithelial cancer cell line (SiHa) in the crude extract of *Vibrio diabolicus* A1SM3 which agrees with the cytotoxic activity previously reported for this compound [27]. Additionally, antibacterial activity against *Staphylococcus aureus* reported for isotrisindoline [6] suggest that the antibacterial activity previously described against *S. aureus* methicillin-resistant (MRSA) by the crude extract of *Vibrio diabolicus* A1SM3 can be also attributed to this compound. This bioactive compound was produced mainly in growth media containing peptone without distinguishing the salinity of the growth media. However, it was not possible to correlate its production with the growth of the strain. As it was evidenced in the media with the peptone only, *Vibrio diabolicus* A1SM3 showed the higher growth with one of the highest isotrisindoline production compared to the growth of the strain in starch and peptone media which showed one of the lowest growths (excluding the media with glucose) but with the major isotrisindoline production.

The indole derivatives have been commonly isolated from marine organisms, plants and bacteria [28], among them, the bis-indolylmethane moiety has a relevant interest in pharmacological applications [8] and a variety of biological activities have been described for this type of molecules as antifungal and antibacterial agents, as immunomodulators, in leukemia therapy and as anticancer agents [8]. So far, related to the biosynthesis of both isomers (trisindoline and isotrisindoline), it has been considered that at least one of the indole moieties is incorporated in post-biosynthetic reactions [5]. It has been described the role of indole as an intercellular signaling molecule that controls various bacterial phenotypes as biofilm formation and other virulence factors in Gram-positive and Gram-negative bacteria, including some species of *Vibrio* [29]. The indole is produced during the stationary cell growth phase and is synthesized from tryptophan by the enzyme tryptophanase encoded by the *tnaA* gene [30]. In addition, this gene is repressed when the level of tryptophan is low [31], which agree with our results of isotrisindoline production in growth media with nitrogen sources rich in tryptophan, as peptone and yeast extract, compared with the non-production of this compound in the cultures grown with casamino acids. This latter nitrogen source is characterized by its low content of tryptophan due to the acid treatment during casein digestion to its production, which destroys the tryptophan [32]. This suggests the biosynthesis of trisindoline isomers requires a source of indole moiety which is synthesized from exogenous tryptophan available in the growth media which was reported by Kwon and Weiss (2009) in the isotrisindoline production by *E. coli* during anaerobic growth [33]. Additionally, the same authors determined that *tnaA* gene is required for the biosynthesis of isotrisindoline by the study of its production in a *tnaA* mutant [33].

Moreover, previous studies have been published regarding the *ipoA* gene involved in the initial oxidation of indole to indoxyl which, in equilibrium, generates 3-oxyindole that could be oxidized again to produce isatin, which has been proposed as the precursor of trisindoline and isotrisindoline by non-enzymatic reaction [34,35].

On the other hand, the studies conducted with the metagenome of the marine sponge *Discodermia calyx* showed that the gene corresponding to ORF 25, homologous to inosine 5′-monophosphate dehydrogenase (IMPD) was necessary for isotrisindoline production by *E. coli* [20]. Takeshige et al. (2015) proposed the IMPD homolog as the responsible for the oxidation of the 3-oxyindole to isatin for further non-enzymatic addition of the two indole moieties to produce isotrisindoline and trisindoline. The annotation of the genome of *Vibrio diabolicus* A1SM3 recently studied in our group (data not published yet) showed the presence of *tnaA* gene responsible for the conversion of tryptophan to indole and the IMPD homolog which putatively produce the isatin. However, the *ipoA* gene responsible for the oxidation of indole to 3-oxyindole was not annotated, this could be related with the high divergence of the strain where it was characterized (*Rhodococcus* sp. strain T104) and *Vibrio diabolicus* A1SM3.

In regard to the results obtained for PHB production in the different modified growth media, agree with previous studies where the addition of complex nitrogen sources (such as casamino acids) in the culture media, promote the PHB production by recombinant *Escherichia coli* strains and *Ralstonia eutropha* DSM 11348 [36,37,38]. This behavior has been explained by the amino acids and peptides composition of this source which facilitate the synthesis of proteins required for the cell functioning reducing the energy cost for the cell to synthesize them [39,40]. Additionally, the result regarding the lower PHB production in media containing glucose was also reported by Chien et al. (2007) [26] who studied the effect of different carbon sources on the PHB production by a marine *Vibrio* spp. finding the lower growth and production in the media containing glucose compared with glycerol, sucrose, sodium acetate, and sodium succinate as carbon sources.

In conclusion, the approach implemented based on the OSMAC strategy coupled with HPLC-MS/MS and the GNPS platform guided the isotrisindoline identification, a bioactive compound with cytotoxic potential, and the PHB, which have been widely described and studied for their promising applications in biotechnology as an alternative solution to replace plastic due to their biodegradability properties [9]. Furthermore, the strategy employed in this work led to establishing the best nutrient sources of the culture medium to enhance these metabolites production under the conditions tested. Additionally, the articulation of these approaches represents a huge advantage in the processing and analysis of the data acquired, which allows going further in metabolites identification. Also, this study allowed determining how the variation in growth conditions, like carbon and nitrogen sources, impact the production of certain compounds. Therefore, these strategies could be also employed to study a range of environmental conditions, such as temperature, pH, co-culture, exposure to exogenous compounds, among others.

## 4. Materials and Methods

### 4.1. Vibrio diabolicus A1SM3 Strain: Crude Extract Production and Fractionation

*Vibrio diabolicus* A1SM3 was isolated from a sediment sample recovered from a Solar Saltern in Manaure, La Guajira, Colombia. This strain was deposited in the Collection of Microorganisms of Universidad de La Sabana (USAB-BIO, Chia, Colombia) registered in the RNC Colombia. The bacteria was seed in growth medium (referred as M3 medium) with the following composition: 40 g/L NaCl, 20 g/L MgSO_4_ 7H_2_O, 1 g/L KCl, 0.3 g/L KH_2_PO_4_, 0.5 g/L yeast extract, 0.5 g/L peptone (pep), 0.5 g/L casamino acids (cas), 0.5 g/L glucose (glu), 0.5 g/L starch (star), and 0.3 g/L sodium pyruvate (pyr) [41]. After 15 days of incubation at 30 °C and 150 rpm, 4L of culture broth was extracted twice with ethyl acetate (1:1) and the organic fraction was concentrated in a rotary evaporator under vacuum. The extract (110 mg) was fractionated by SiO_2_ column chromatography (250 × 15 mm) using n-hexane:ethyl acetate (7:3) to ethyl acetate: methanol (9:1) gradient. A total of 80 fractions were obtained and grouped in 15 subfractions according to their TLC profiles. The grouped fractions were tested for cytotoxic activities and analyzed by HPLC-MS/MS and NMR (Bruker Ascend^TM^ 600 MHz, Bruker Daltonics, Billerica, MA, USA).

### 4.2. Cytotoxic Activity

The fractions were evaluated for their cytotoxic activity against two human cancer cell lines and one non-tumor cell line by MTT cell proliferation assay [42]. SiHa, human cervix epithelial cells (ATCC^®^ HTB-35 ^TM^) and human lung cell line A549 (ATCC^®^ CRM-CCL-185 ^TM^) were grown in Dulbecco’s modified Eagle’s medium—DMEM (Sigma-Aldrich Co., Darmstadt, Germany) supplemented with antibiotic agents (penicillin 120 IU/mL and streptomycin 100 IU/mL, Gibco/Invitrogen, Paisley, UK) and 10% fetal bovine serum (Eurobio, Les Ulis, France). L929, Fibroblasts (ATCC^®^ CCL-1 ™) were incubated in RPMI medium supplemented with 1% (v/v) L-glutamine (Sigma-Aldrich Co., Darmstadt, Germany), 10% (v/v) fetal bovine serum (Eurobio, Les Ulis, France), 1% (v/v) penicillin and 1% (v/v) streptomycin (Gibco/Invitrogen, Paisley, UK). L929 was used as a non-tumor cell line for toxicity control. The cells were maintained in a humidified atmosphere of 5% CO_2_ at 37 °C. Doxorubicin was used as a positive control [43]. For MTT assay, 1 × 10^5^ cells were gently placed into each well of a 96-well plate. After cell adhesion, the culture medium was changed for fresh medium containing different concentrations of the fractions (10, 25, 50, 80, and 100 μg/mL) diluted in less than 0.5% of DMSO and the cells were kept at 37 °C with 5% CO_2_ for 72 h. Cell viability was measured by MTT colorimetric assay and 50% inhibitory concentration (IC_50_) was calculated [44]. Negative controls for DMSO toxicity was also evaluated at 0.5%. Statistical analysis was performed using GraphPad Prism 6^®^ and Microsoft^®^ Excel 2016. All the experiments were repeated at least three times and the results were expressed as mean values ± standard deviation. IC_50_ values were obtained by nonlinear regression.

### 4.3. Modified Culture Media and Metabolite Extraction of Vibrio diabolicus A1SM3 Cultures

The strain was cultured in different modified culture media varying the carbon and nitrogen source according to the initial growth medium composition, having into account that the total amount of the nutrient components was kept constant when the carbon and nitrogen sources of the modified media components were changed [45]. A binary mixture was chosen to evaluate the effects of the three carbon sources: glucose (Merck, Darmstadt, Germany), sodium pyruvate (Fisher Scientific, Pittsburgh, PA, USA) and starch (Carlo Erba, Val de Reuil, France) combined with the three nitrogen sources: peptone (Oxoid, Basingstoke, UK), yeast extract (Oxoid, Basingstoke, UK) and casamino acids (AMRESCO Inc, VWR International, LLC; OH, USA) based on the initial composition of M3 medium. The modified media consisted of 12 combinations (Table 1) including three experiments with only one nitrogen source (peptone, casamino acids or yeast extract) as a unique nutrient source and nine binary mixtures. Additionally, replicates of culture in M3 medium and experiments *pyr_cas*, *cas*, *yeast,* and *pep* (as named in Table 1) were done. All growth media also contained 20 g/L MgSO_4_·7H_2_O, 1 g/L KCl and 0.3 g/L KH_2_PO_4_ and NaCl according to the salinity tested (10, 40, and 70 g/L). The cultures (30 mL) were incubated for 15 days at 30 °C and 150 rpm. Samples from the cultures were taken every 24 h until day 4, after that, samples were taken in day 6, 11 and 15 to measure bacterial growth by OD_595_ and to calculate the area under the curve (AUC) for each culture. These results were analyzed using two-way ANOVA and Tukey HSD test for comparison of means in R software [46]. The statistical significance was determined at *p* values < 0.05. After that, the whole culture broth was extracted twice with ethyl acetate (1:1) and the organic fraction was subjected to rotary evaporation under vacuum. A solution of 1 mg/mL of each extract in acetonitrile-methanol (7:3) was prepared for further HPLC-MS/MS analysis. The non-inoculated media were also extracted and used as a control.

### 4.4. HPLC-MS/MS Analysis

Twenty μL (1 mg/mL) of crude extracts and fractions were analyzed by HPLC-MS/MS using a Shimadzu system (Shimadzu, Tokyo, Japan), coupled with a micrOTOF-Q II mass spectrometer (Bruker Daltonics, Boston, MA, USA) equipped with an ESI source and a quadrupole-time of flight analyzer (qTOF, Bruker Daltonics Inc., Billerica, MA, USA). For chromatographic analyses it was employed a Kinetex C18 column (2.6 µm, 150 × 4.6 mm) (Phenomenex, Torrance, CA, USA) kept at 40 °C, with a flow rate of 0.8 mL/min applying a gradient solvent with phase A (water with 0.1% formic acid) and phase B (acetonitrile with 0.1% formic acid), from 10% to 40% of B in 6 min, to 70% of B in 12 min, and to 100% of B in 22 min. The MS data were acquired in positive mode using an MS range of *m*/*z* 50–1300. The equipment was calibrated with trifluoroacetic acid (TFA) every day, and internally during each run. The MS parameters were established as follows: nebulizer gas pressure, 4.5 Bar; dry gas flow, 9 L/min; capillary voltage, 3500 V; ion source temperature, 220 °C; spectra rate acquisition, 2 spectra/s. Auto MS/MS fragmentation was carried out for the four most intense ions per spectrum, and it was performed applying a gradient of collision-induced dissociation energy from 20 to 105 eV according to the parent mass. In addition, the precursor ion was released after the acquisition of MS/MS spectra. All MS data were analyzed with Bruker Compass DataAnalysis 4.3 software (Bruker Daltonics, Boston, MA, USA).

### 4.5. HPLC-MS Data Processing

The converted MS data from the modified culture media and fractions were imported to MZmine 2.30 software (VTT Technical Research Center, Helsinki, Finland and Turku Center for Biotechnology, Turku, Finland) [47] and processed following these steps; mass detection, chromatogram builder, chromatogram deconvolution, deisotoping, normalization, and alignment [48,49,50]. The data processing parameters are shown in Supplementary Table S4. The aligned peak list was exported to CSV format with the peak areas of each feature. All features from solvent blanks were excluded. In addition, dereplication of the bioactive fractions were made using Antibase and MarinLit (http://pubs.rsc. org/marinlit/) databases.

### 4.6. MS/MS Data Processing for Molecular Networking

The raw data from HPLC-MS/MS was used to create a molecular network. The MS/MS data were converted to mzXML files using the MSConvert from ProteoWizard software (ProteoWizard, Palo Alto, CA, USA) [51] and then uploaded into the Global Natural Products Social Molecular Networking web-platform (http://gnps.ucsd.edu) [16]. A molecular network was created using the online workflow at GNPS. The data were filtered by removing all MS/MS peaks within ±17 Da of the precursor *m*/*z*. The data were then clustered with MS-Cluster with a parent mass tolerance of 0.02 Da and an MS/MS fragment ion tolerance of 0.02 Da to create consensus spectra. Furthermore, consensus spectra that contained less than two spectra were discarded. A network was then created where edges were filtered to have a cosine score above 0.7 and more than four matched peaks. Further edges between two nodes were kept in the network if and only if each of the nodes appeared in each other’s respective top 10 most similar nodes. The spectra in the network were then searched against GNPS spectral libraries. The library spectra were filtered in the same way as the input data. All matches kept between network spectra and library spectra were required to have a score above 0.7 and at least six matched peaks. For visualization and more specific analysis, the network data was exported and analyzed into Cytoscape (Version 3.6, Cytoscape consortium, San Diego, CA, USA) [21]. Two networks were constructed, the first one included the 36 extracts from modified culture media in the three salinities tested plus the 15 fractions from *Vibrio diabolicus* A1SM3, and in the second one, the 15 fractions were excluded to analyze the variation of metabolites production in the extracts from modified culture media.

### 4.7. NMR Analysis

The NMR experiments were carried out in a Bruker Avance III spectrometer using a 14.1 Tesla, (600.12 MHz in hydrogen frequency) magnetic field and 3 mm inverse triple cryoprobe ^1^H, ^13^C, and ^15^N nuclei. Each fraction was solubilized using 180 µL of CD_3_CN (Sigma-Aldrich Co., Darmstadt, Germany). Chemical shifts to all experiments were calibrated from the solvent signal (CD_3_CN) at 1.94 ppm. ^1^H experiments were performed using the zg30 pulse sequence. Instrumental parameters were set up as follow: 1.0 s relaxation delay, spectral width from 0 to 12 ppm, 32 k points, and 2.72 s of acquisition time. The post-processing was performed using a 0.1 Hz (Line Broadening—LB) exponential multiplication factor, and phase and baseline were manually corrected. ^1^H-^13^C HMBC experiments (hmbcetgpl3nd) were carried out using a spectral width of 0 to 10 ppm for the F2 dimension (^1^H) and 0 to 200 ppm for the F1 dimension (^13^C). An acquisition time of 0.0852 s, relaxation delay 2 s, and 32 scans for 128 increments was employed. Mono dimensional ^1^H TOCSY (seldigpzs) experiments were performed at 7.18, 6.909, and 6.86 ppm. Regarding these experiments, the following conditions were used: spectral width of 0 to 10 ppm, 2.72 s acquisition time, 1.0 s relaxation delay, 32 k points, and 128 scans.

### 4.8. Statistical Analysis

The resulting table from the MZmine processing was used here. The matrix data were scaling by Pareto algorithm prior to multivariate analyses. Subsequent principal component analysis (PCA) and hierarchical clustering analysis were built using the R software version 3.3.1 employing the *factoextra* [52] and *dendextend* [53] packages, respectively.

## Figures and Tables

**Table 1:** Composition of each nutrient source in the modified culture media and their coded

Modified Culture Media	Composition of Nutrient Sources* (g/L)					
	Glucose	Starch	Sodium Pyruvate	Yeast Extract	Peptone	Casamino Acids
pyr_cas	0.0	0.0	1.3	0.0	0.0	1.5
pyr_pep	0.0	0.0	1.3	0.0	1.5	0.0
pyr_yeast	0.0	0.0	1.3	1.5	0.0	0.0
star_cas	0.0	1.3	0.0	0.0	0.0	1.5
star_pep	0.0	1.3	0.0	0.0	1.5	0.0
star_yeast	0.0	1.3	0.0	1.5	0.0	0.0
glu_cas	1.3	0.0	0.0	0.0	0.0	1.5
glu_pep	1.3	0.0	0.0	0.0	1.5	0.0
glu_yeast	1.3	0.0	0.0	1.5	0.0	0.0
cas	0.0	0.0	0.0	0.0	0.0	2.8
pep	0.0	0.0	0.0	0.0	2.8	0.0
yeast	0.0	0.0	0.0	2.8	0.0	0.0

* The composition is expressed as g/L. The total amount of nutrient sources was kept at 2.8 g/L, therefore, the relative proportion of carbon sources correspond to 46% (1.3 g/L) and for nitrogen sources was 54% (1.5 g/L). Glucose (glu), sodium pyruvate (pyr), starch (star), peptone (pep), casamino acids (cas), and yeast.

## Supplementary Materials

The following are available online at https://www.mdpi.com/1660-3397/17/4/196/s1, Table S1. PCA coefficients for the variables; Table S2. Fractionation of crude extract and cytotoxic activity from the fractions of *Vibrio diabolicus* A1SM3 grown in M3 medium; Table S3. NMR data of isotrisindoline measured in CD_3_CN-d3 (1H: 600 MHz; 13C: 150 MHz); Table S4. MZmine 2.30 processing parameters; Figure S1. Bacterial growth of *Vibrio diabolicus* A1SM3 in the modified growth media; Figure S2. Chromatograms of the cytotoxic fractions of *Vibrio diabolicus* A1SM3 with IC_50_ values; Figure S3. MS/MS spectrum for isotrisindoline in fraction F5; Figure S4. (a) HSQC and (b) HMBC of F5 fraction measured in CD_3_CN-d^3^ (^1^H: 600 MHz; ^13^C: 150 MHz).
