# Supplementary material for: Integrating Molecular Network and Culture Media Variation to Explore the Production of Bioactive Metabolites by Vibrio diabolicus A1SM3
Source: Mar Drugs. 2019 Mar 27;17(4):196. doi: 10.3390/md17040196 (PMC6520778; doi:10.3390/md17040196)
Supplement: Supplementary file 1 [file marinedrugs-17-00196-s001.pdf]

marine drugs

Supplementary Materials

Integrating molecular network and culture media variation to explore the production of bioactive metabolites by *Vibrio diabolicus* A1SM3

Natalia Conde-Martínez [1], Anelize Bauermeister [2,3], Alan Cesar Pilon [2], Norberto Peporine Lopes [2] and Edisson Tello [1,*]

1 Bioscience Doctoral Program, Grupo de Investigación en Bioprospección, Faculty of Engineering, Universidad de La Sabana, Campus Puente del Común, Km 7, Autopista Norte de Bogotá, 250001, Chía, Colombia; nataliacoma@unisabana.edu.co

2 Núcleo de Pesquisa em Produtos Naturais e Sintéticos (NPPNS), Faculdade de Ciências Farmacêuticas de Ribeirão Preto, Universidade de São Paulo, 14040-903 Ribeirão Preto/SP, Brazil; ane_qui@hotmail.com (A.B.); pilonac@gmail.com (A.C.P.); npelopes@fcfrp.usp.br (N.P.L.)

3 Departamento de Farmacologia, Instituto de Ciências Biomédicas, Universidade de São Paulo, 05508-900 São Paulo-SP, Brazil

* Correspondence: edisson.tello@unisabana.edu.co; Tel.: 57-(1)-8615555 (Ext: 25219)

marine drugs

Table S1. PCA coefficients for the variables in the 10 principal components.

	PC1	PC2	PC3	PC4	PC5	PC6	PC7	PC8	PC9	PC10
VIB_CAS_1	-5.7439	2.6141	-1.359	6.7812	-1.7709	1.1415	-2.6509	0.4078	-0.9579	0.0762
VIB_CAS_4	-7.4338	3.4806	-5.7088	7.6803	-2.6359	1.8873	-4.016	2.2199	-2.9821	0.0491
VIB_CAS_7	-2.1277	1.4339	4.2429	0.7956	-0.5252	-1.2179	0.1	-4.9625	2.2316	0.9228
VIB_GLU_CAS_1	-9.1492	4.9993	-15.4724	-0.3371	-0.5641	0.7097	-2.4206	2.3191	-9.0348	-5.2999
VIB_GLU_CAS_4	-16.3362	9.0604	-49.4769	-23.2261	3.2101	-2.7542	5.8603	-2.7288	6.4727	2.8525
VIB_GLU_CAS_7	-6.2318	2.6775	-3.9053	4.7924	-0.9516	1.1285	-1.6446	0.8653	-0.4835	0.074
VIB_GLU_PEP_1	-0.0482	-1.9648	7.4544	-6.5313	13.6193	3.9074	5.8826	3.5663	-4.702	-2.5689
VIB_GLU_PEP_4	-0.0967	-2.3693	8.3629	-7.4576	16.8054	4.2325	7.1721	4.6115	-6.7993	-3.7672
VIB_GLU_PEP_7	-2.4776	0.3828	4.1105	0.7916	4.7136	1.2269	2.2345	0.5311	1.3871	0.9845
VIB_GLU_YEAST_1	-4.1292	0.8673	2.4539	5.0455	0.079	0.8885	0.5926	-0.8054	4.7922	2.3944
VIB_GLU_YEAST_4	-4.5341	0.9934	1.9617	5.5787	0.0059	1.391	0.2028	-0.8117	3.9445	2.8791
VIB_GLU_YEAST_7	-6.5489	2.5228	-2.3945	7.7646	-1.6162	1.9219	-2.6529	0.8998	-0.8012	0.5174
VIB_M3_1	23.8515	3.4415	1.8441	-3.4679	-17.2023	-13.6931	17.8214	-1.0341	-14.4761	13.261
VIB_M3_4	-0.0204	0.9628	7.5389	-4.7089	-2.4039	-2.0916	3.4026	-26.9919	-4.0187	-14.1368
VIB_M3_7	-1.5938	0.5965	5.013	-0.9591	-0.6981	-0.7107	0.7526	-3.9618	1.4835	0.3603
VIB_PEP_1	1.1507	-1.2055	13.7931	-25.8704	-22.9004	18.9448	-6.4934	7.0166	2.7295	-3.148
VIB_PEP_4	-2.6642	0.2775	5.041	-0.4723	-1.2563	1.2514	1.2844	-2.5183	2.4502	2.8168
VIB_PEP_7	-1.4818	-0.0023	8.783	-8.8311	-4.8633	5.1803	0.0727	-1.5399	1.9107	1.1325
VIB_PYR_CAS_1	-5.2435	2.1632	-1.3923	4.6853	-0.9651	0.917	-1.1686	-0.2244	0.9491	0.5626
VIB_PYR_CAS_4	-2.6505	2.1356	1.8922	1.0914	-0.7912	-4.1273	-4.7348	-1.0861	0.5952	-0.6383
VIB_PYR_CAS_7	-8.7068	4.7577	-10.9204	6.517	-4.0523	1.667	-7.2537	4.4256	-15.4672	-7.4771
VIB_PYR_PEP_1	-3.9401	1.1186	2.6773	4.3703	0.2793	0.2796	0.9265	-1.8585	4.664	2.7019
VIB_PYR_PEP_4	-2.4355	0.5724	4.9539	-0.121	1.8569	0.6015	1.7456	-2.767	2.0944	1.1442
VIB_PYR_PEP_7	-6.8106	2.8891	-3.4867	7.5333	-2.1013	1.6304	-3.2254	1.4319	-1.5971	0.4501
VIB_PYR_YEAST_1	-4.2034	1.1283	2.4188	5.1892	-0.4351	0.9546	0.3254	-1.2439	4.7406	2.8567
VIB_PYR_YEAST_4	-6.8943	2.9436	-2.9082	9.2583	-2.9674	2.3029	-4.0843	1.9878	-1.8597	0.9968
VIB_PYR_YEAST_7	-7.6281	3.2204	-4.8226	9.2368	-3.2592	2.5801	-4.3932	2.8474	-3.4651	0.0987
VIB_STAR_CAS_1	71.6993	16.9427	-13.7742	3.395	6.9206	11.1208	-9.005	-2.8848	1.6183	0.8458
VIB_STAR_CAS_4	18.4288	4.4041	0.2312	1.9052	-5.6719	-17.2395	10.0685	13.9373	8.4546	-18.0979
VIB_STAR_CAS_7	2.7474	0.4688	10.6052	-15.3313	4.586	-25.7735	-25.3736	0.3303	-0.427	3.7919
VIB_STAR_PEP_1	-0.9789	-0.5348	7.2167	-5.2065	7.5776	1.9502	3.7497	1.4927	-1.6606	1.0593
VIB_STAR_PEP_4	-1.1381	-1.4219	7.1503	-4.6828	10.8054	2.617	4.2909	3.4583	-1.4746	1.0777
VIB_STAR_PEP_7	-0.7362	-2.4509	9.2728	-9.5224	10.6578	1.338	2.5567	4.5433	-3.9074	3.3517
VIB_STAR_YEAST_1	-3.1458	0.0928	3.1313	3.222	2.2931	0.4976	1.289	-0.2513	3.0196	2.3933
VIB_STAR_YEAST_4	-3.9444	1.108	2.39	4.2505	0.4195	0.2902	0.6084	-1.5014	4.2583	2.303
VIB_STAR_YEAST_7	13.3619	-71.8045	-12.7836	2.3279	-1.3605	0.2324	-2.0474	-0.5535	0.0018	-0.4538
VIB_YEAST_1	-0.8445	0.9684	2.3266	4.2955	-1.3992	-1.5087	3.4075	-0.3076	5.3416	1.9336
VIB_YEAST_4	-4.3529	1.3267	1.8368	5.0585	-0.0583	0.6105	0.2376	-0.9515	3.8299	2.0238
VIB_YEAST_7	3.0318	1.203	1.7027	5.1596	-3.3798	-4.2848	6.5802	2.0926	7.1449	-0.3238

marine drugs

Table S2. Fractionation of crude extract and cytotoxic activity from the fractions of *Vibrio diabolicus* A1SM3 grown in M3 medium.

Fraction	Weight (mg)	Cytotoxic activity IC_{50} (µg/mL)		
		**SiHa**	**A549**	**L929**
F1	12.4	>100	>100	>100
F2	3.5	>100	>100	>100
F3	27.0	>100	>100	>100
F4	2.6	>100	>100	>100
**F5**	**3.7**	**28**	**>100**	**>100**
**F6**	**3.3**	**80**	**>100**	**>100**
F7	1.7	NT*	NT*	NT*
F8	1.4	NT*	NT*	NT*
F9	9.0	>100	>100	>100
F10	1.1	NT*	NT*	NT*
F11	5.3	>100	>100	>100
F12	4.2	>100	>100	>100
F13	9.2	>100	>100	>100
F14	5.2	>100	>100	>100
F15	3.3	>100	>100	>100

* NT. Not tested

marine drugs

MDPI

Table S3. NMR data of isotrisindoline (2,2-di(3-indolyl)-3-indolone) measured in CD3CN-d3 (1H: 600 MHz; 13C: 150 MHz) compared with the NMR data reported in literature and the NMR data of trisindoline.

Position C-H	^{1}H NMR and ^{13}C NMR for isotrisindoline in CD_3CN acquired in this work				^{1}H and ^{13}C NMR for isotrisindoline in $CDCl_3$ *		^{1}H and ^{13}C NMR for isotrisindoline in $CDCl_3$ **		^{1}H and ^{13}C NMR for trisindoline in CD_3OD ***	
	δ_H, Mult	δ_C, HSQC	Mult	HMBC (δ_H to δ_C)	δ_H (ppm)	δ_C (ppm)	δ_H (ppm)	δ_C (ppm)	δ_H (ppm)	δ_C (ppm)
5	6.83, t	119.7	CH	C7, C7a	6.89	119.4	6.87	118.7	6.97	124.5
5'	6.88, t	120.1	CH	C4', C3a'	7.19	119.9	6.93	118.7	6.81	120.3
4	7.57, d	125.9	CH	C3a	7.73	125.5	7.69	124.8	7.27	127.3
6'	7.08, t	122.9	CH	C7', C7a'	7.09	122.3	7.11	121.2	7.03	122.9
2'	7.15, d	124.7	CH	3', 3a', 7a'	6.94	124.1	7.13	124.0	6.90	126.4
7'	7.40, d	112.6	CH	C4', C5', C6'	7.62	111.4	7.37	111.2	7.32	113.1
4'	7.29, d	121.6	CH	C5', C3a'	7.36	120.4	7.40	119.9	7.27	123.2
6	7.54, t	138.4	CH	C7	7.51	137.6	7.52	137.5	7.25	129.8
7	6.99, d	113.4	CH	C2, C6	6.91	112.9	6.94	112.3	7.05	111.8
3'	-	115.0	C			115.1		113.7		116.5
3a	-	119.5	C			120.8		119.0		137.5
3a'	-	126.7	C			125.7		125.3		128.1
7a'	-	138.0	C			137.0		136.9		139.6
7a		161.3				160.3		160.5		143.1
2	-	-	C			68.2		68.2		183.4
3	-	-	C=O			201.5		201.8		55.7

* NMR data obtained from Takeshige, Y.; Egami, Y.; Wakimoto, T.; Abe, I. Production of indole antibiotics induced by exogenous gene derived from sponge metagenomes. Mol. BioSyst. 2015, 11, 1290–1294.
** NMR data obtained from Bell, R.; Carmeli, S.; Sar, N. Vibrindole A, a Metabolite of the Marine Bacterium, *Vibrio parahaemolyticus*, Isolated from the Toxic Mucus of the Boxfish Ostracion cubicus. J. Nat. Prod. 1994, 57, 1587–1590.
*** NMR data obtained from Yoo, M.; Choi, S.-U.; Choi, K.Y.; Yon, G.H.; Chae, J.-C.; Kim, D.; Zylstra, G.J.; Kim, E. Trisindoline synthesis and anticancer activity. Biochem. Biophys. Res. Commun. 2008, 376, 96–99.

marine drugs

Table S4. MZmine 2.30 processing parameters.

Step	Parameter	Value
Baseline corrector	*m*/*z* bin width	1.0000
	Smoothing	2
	Asymmetry	0.001
Mass detection	Mass detector	Centroid
	Noise level	1.0E4
Chromatogram builder	Min time span (min)	0.07
	Min height	1.0E4
	m/*z* tolerance	0.005
Chromatogram deconvolution	Algorithm	Baseline cut-off
	Min peak height	1.0E4
	Peak duration range (min)	0.04 - 3.00
	Baseline level	1.1E4
Deisotoping	*m*/*z* tolerance	0.005
	Retention time tolerance (min)	0.3
	Maximum charge	1
	Representative isotope	Most intense
Normalization	Linear normalizer	Total raw signal peak area
Alignment	algorithm	Join aligner
	m/*z* tolerance	0.005
	Weight for *m*/*z*	20
	Retention time tolerance	0.5
	Weight for RT	10

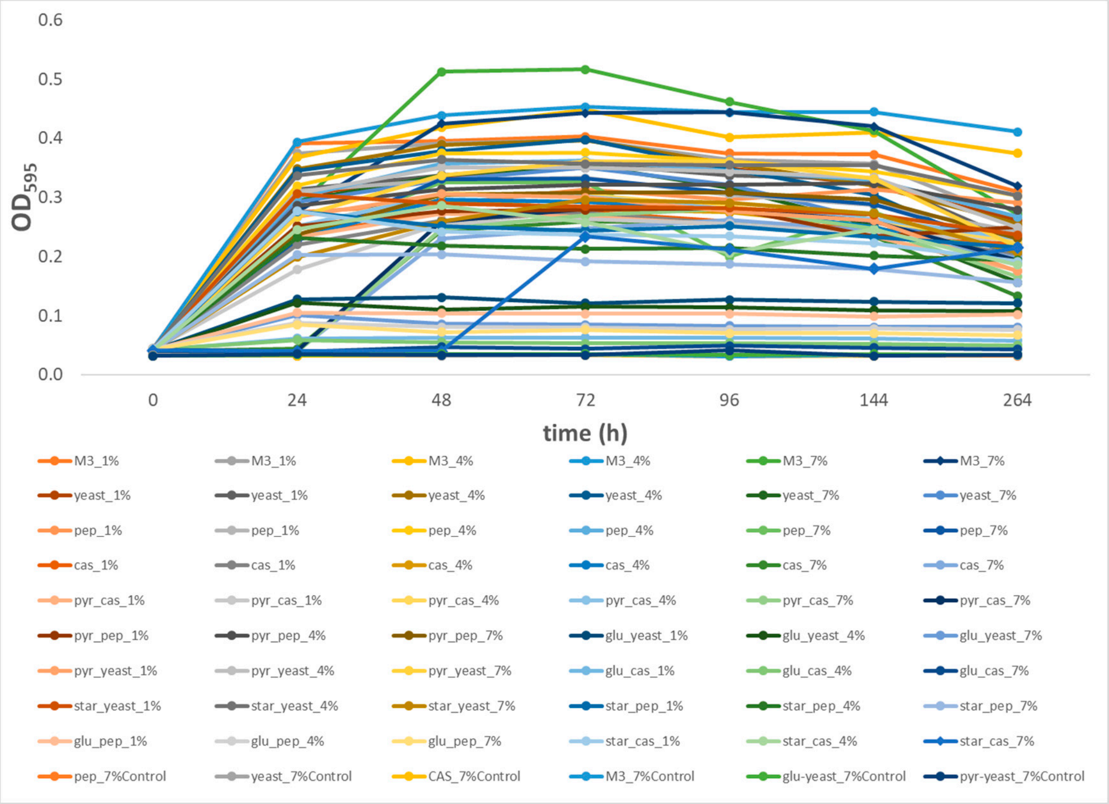

Figure S1. Bacterial growth of *Vibrio diabolicus* A1SM3 in the modified growth media.

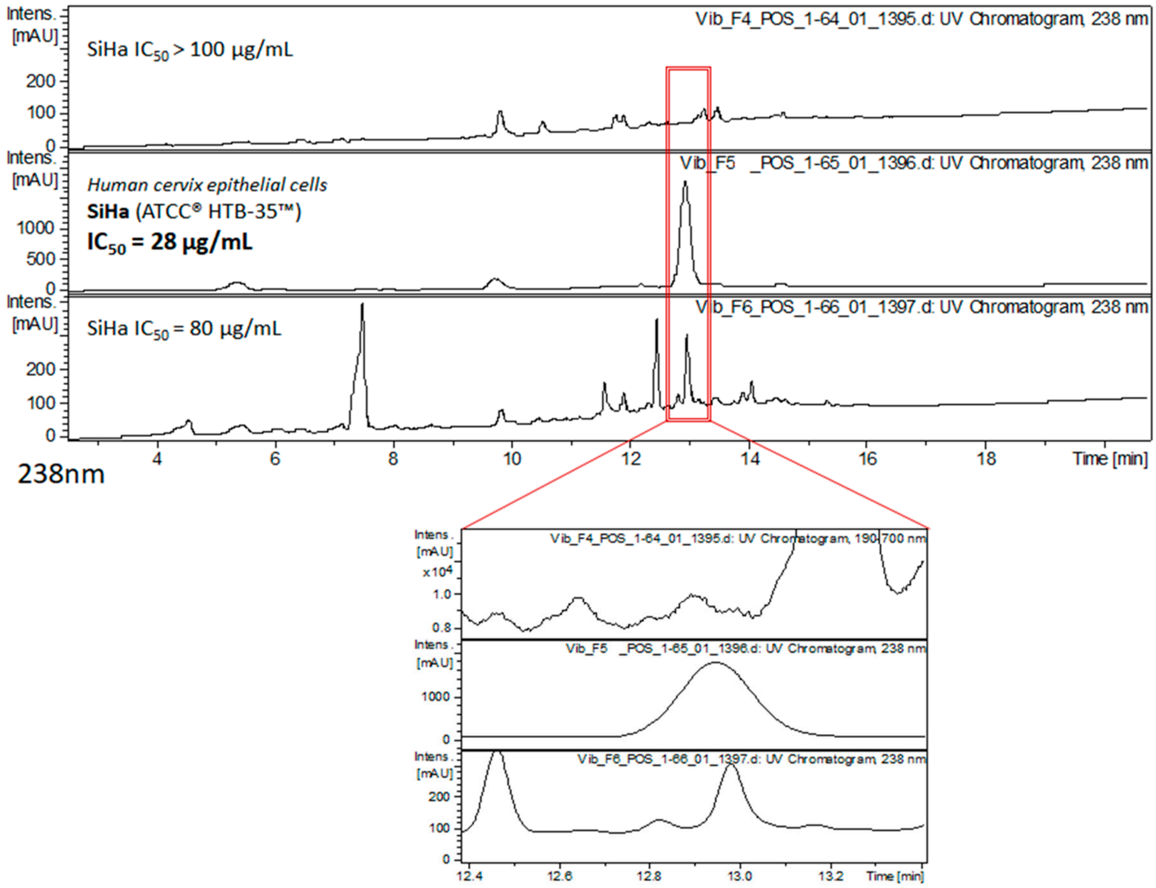

Figure S2. Chromatograms of the cytotoxic fractions of *Vibrio diabolicus* A1SM3 with IC_{50} values.

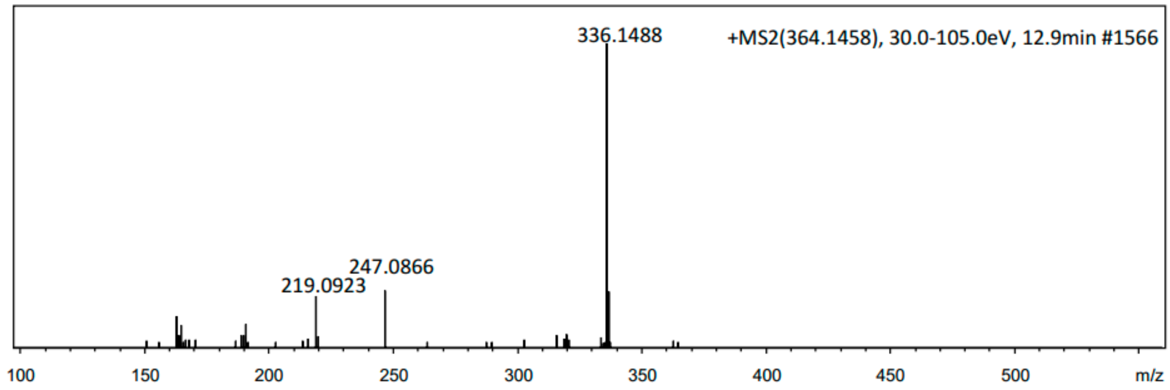

Figure S3. MS/MS spectrum for isotrisindoline in fraction F5.

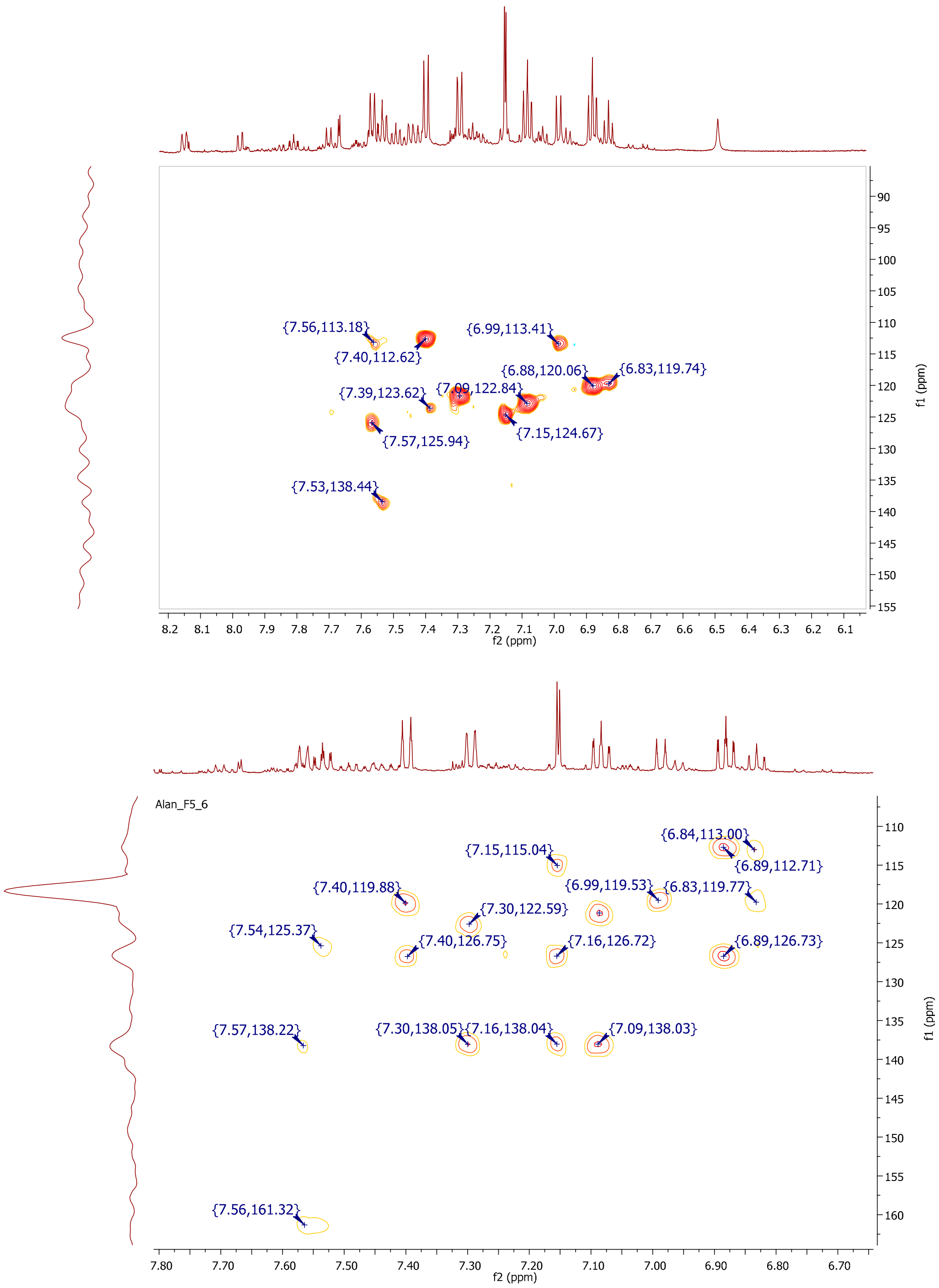

Figure S4. (**a**) HSQC and (**b**) HMBC of F5 fraction measured in CD_3CN-d^3 (^1H: 600 MHz; ^{13}C: 150 MHz).
